# Correction: Cheng et al. Glutathione S-Transferases S1, Z1 and A1 Serve as Prognostic Factors in Glioblastoma and Promote Drug Resistance through Antioxidant Pathways. *Cells* 2022, *11*, 3232

**DOI:** 10.3390/cells12141908

**Published:** 2023-07-21

**Authors:** Bo Cheng, Yu Wang, Abiola Abdulrahman Ayanlaja, Jing Zhu, Piniel Alphayo Kambey, Ziqiang Qiu, Caiyi Zhang, Wei Hu

**Affiliations:** 1 Department of Psychiatry, The Affiliated Xuzhou Eastern Hospital of Xuzhou Medical University, Tongshan Road 379, Xuzhou 221000, China; 301810111019@stu.xzhmu.edu.cn (B.C.); 710020220276@xzhmu.edu.cn (J.Z.); 100002010039@xzhmu.edu.cn (Z.Q.); 2 The Key Lab of Psychiatry, Xuzhou Medical University, Tongshan Road 209, Xuzhou 221000, China; 3 Department of Geriatric Psychiatry, The Affiliated Brain Hospital of Nanjing Medical University, Guangzhou Road 264, Nanjing 220029, China; wangy@stu.njmu.edu.cn; 4 Department of Neurology, Johns Hopkins University School of Medicine, 201 N Broadway, Baltimore, MD 21287, USA; aayanla1@jhmi.edu; 5 Department of Neurobiology and Cell Biology, Xuzhou Medical University, Tongshan Road 209, Xuzhou 221000, China; 502552218104@stu.xzhmu.edu.cn

The authors wish to make the following change to their paper [1]. Figure 7A (marked in red) needs to be corrected. Figure 7 should be changed from:

 
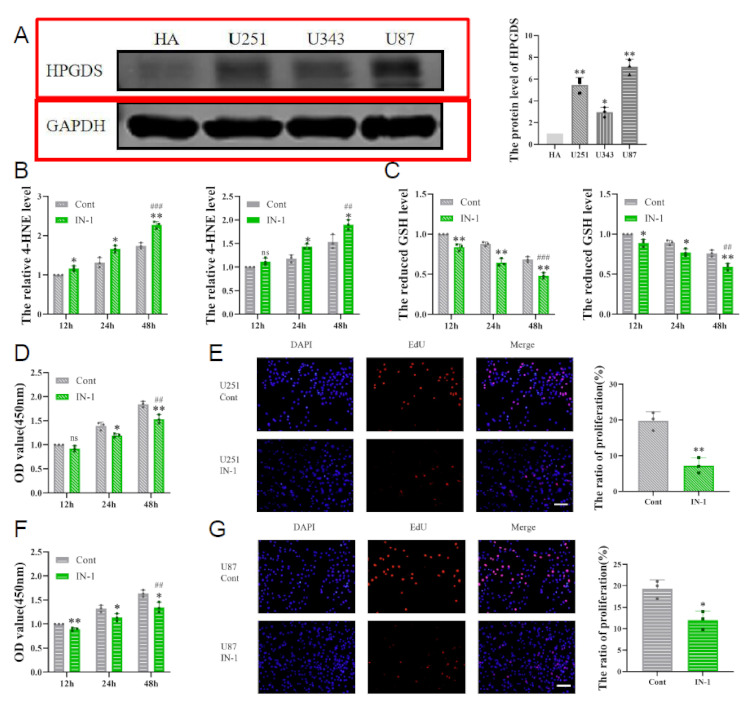


to:

 
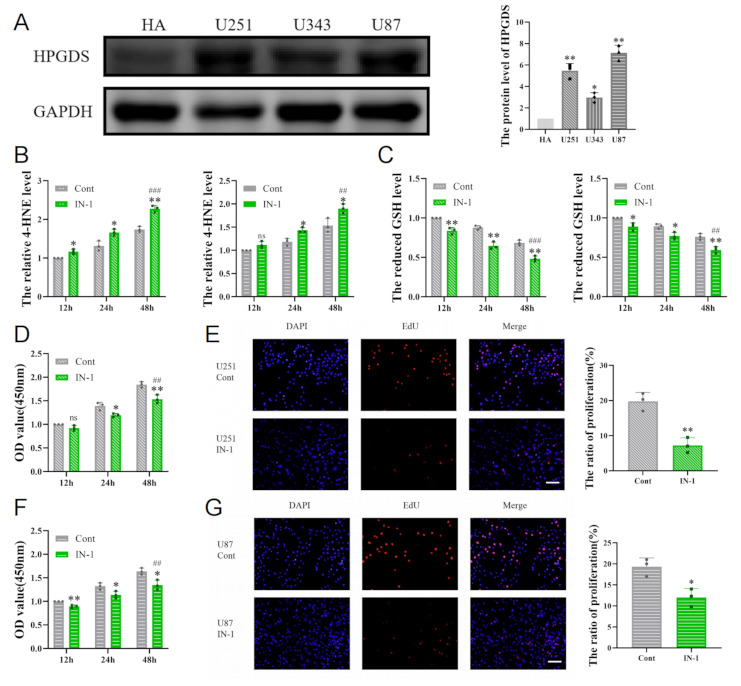


The authors would like to apologize for any inconvenience caused to the readers by these changes. The changes do not affect the scientific results. The original publication has also been updated.
