# Supplementary material for: Glutathione S-Transferases S1, Z1 and A1 Serve as Prognostic Factors in Glioblastoma and Promote Drug Resistance through Antioxidant Pathways
Source: Cells. 2022 Oct 14;11(20):3232. doi: 10.3390/cells11203232 (PMC9600210; doi:10.3390/cells11203232)
Supplement: Supplementary file 1 [file cells-11-03232-s001.zip › cells-1943118-supplementary.pdf]

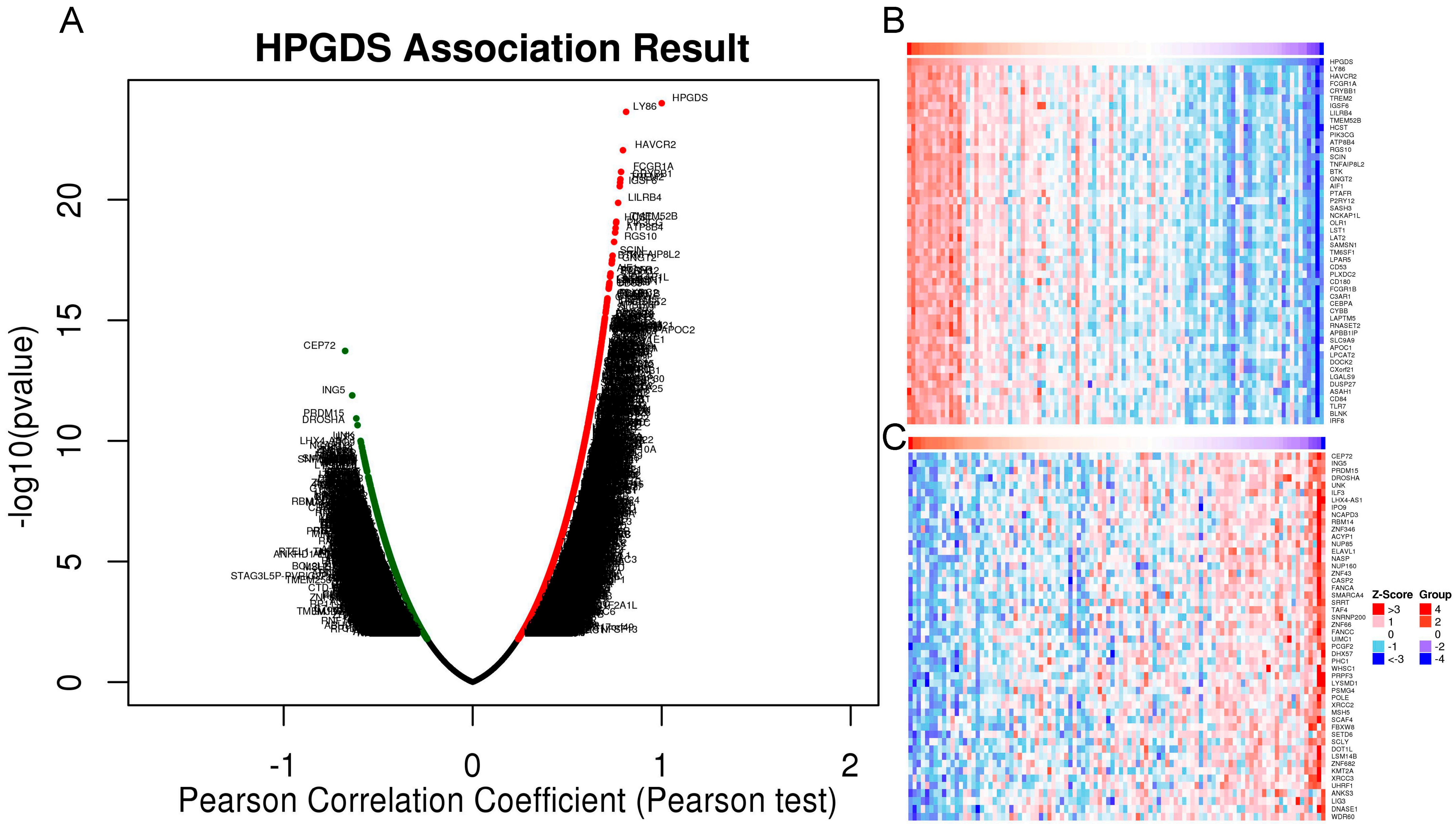


**Figure S1.** Functional enrichment analysis of HPGDS related genes in GBM. (**A**) Based on the TCGA database, we identified the genes with the highest correlation with HPGDS in GBM, and the genes with positive correlation and negative correlation are displayed in (**B**) and (**C**), respectively.

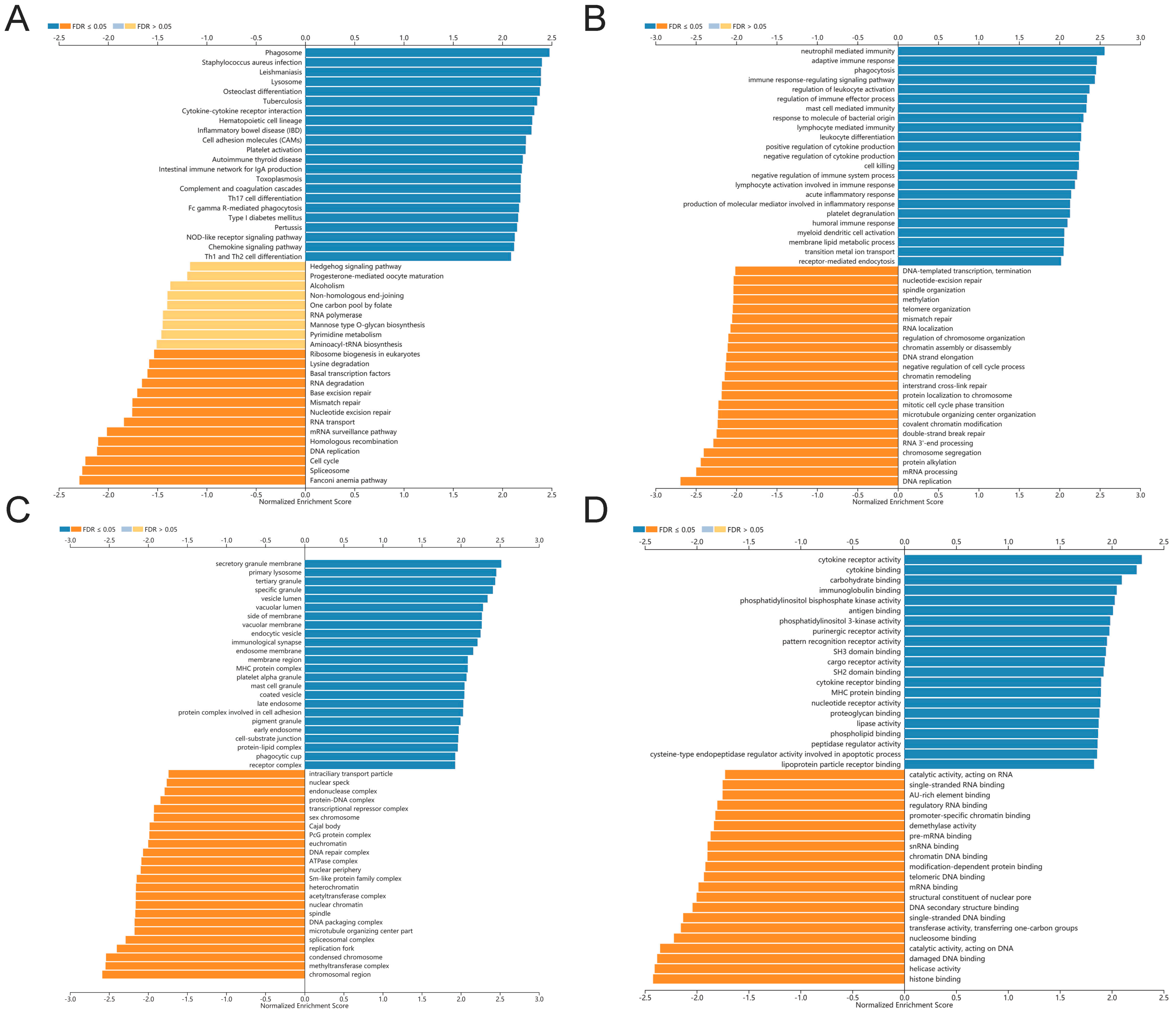


**Figure S2.** GSEA analysis of biological functions of HPGDS in GBM using LinkedOmics. (**A**) KEGG pathway analysis based on GSEA demonstrates the effect of HPGDS expression on signaling pathways in GBM. (**B**–**D**) GO analysis based on GSEA demonstrates the effects of HPGDS expression on cellular components, biological processes and molecular functions in GBM.

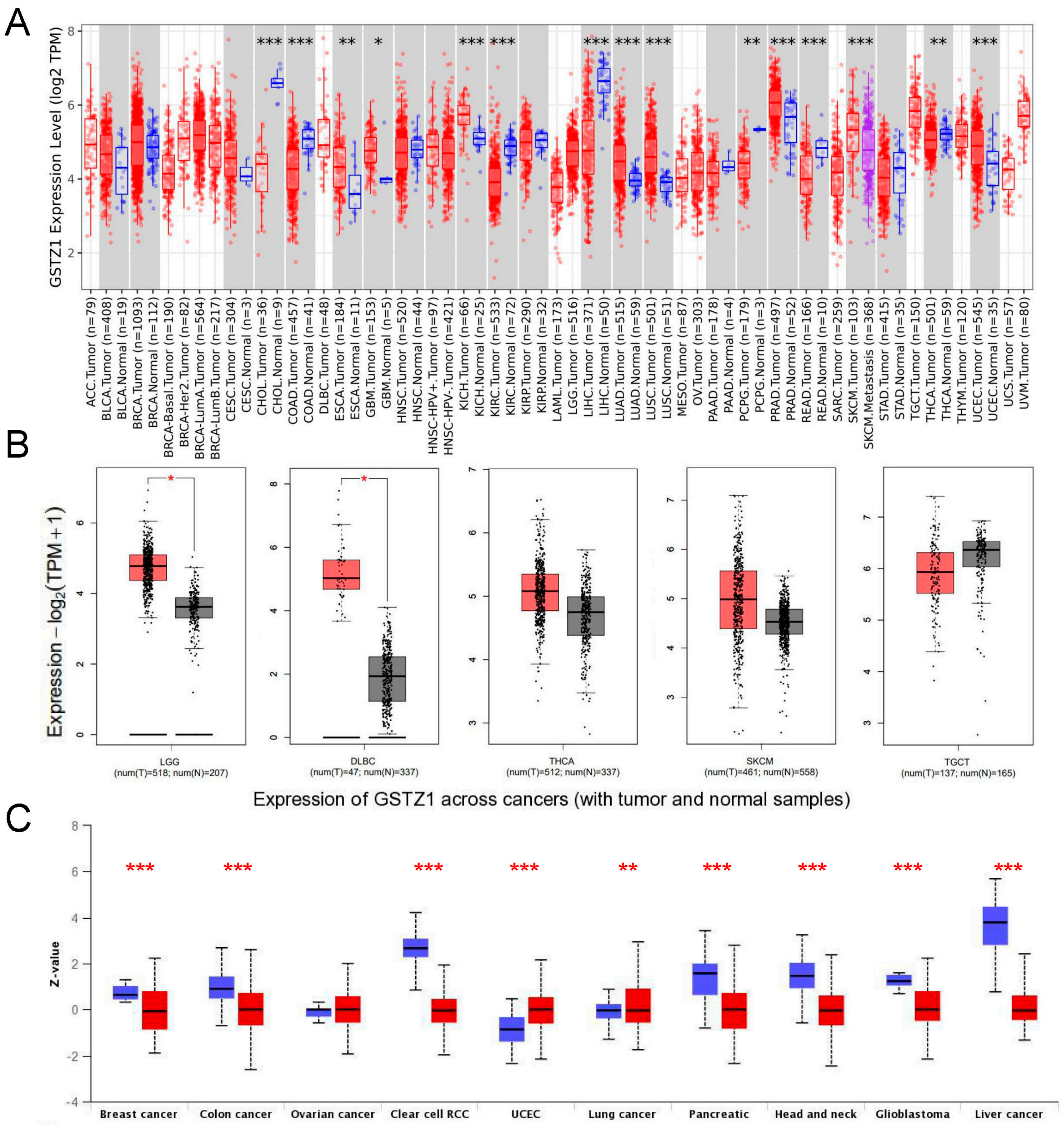


**Figure S3.** The levels of GSTZ1 mRNA and protein in different cancers. (**A**,**B**) The mRNA level of GSTZ1 in different cancers was analyzed with TIMER2. (**C**) The protein level of GSTZ1 in different cancer types was analyzed with UALCAN.

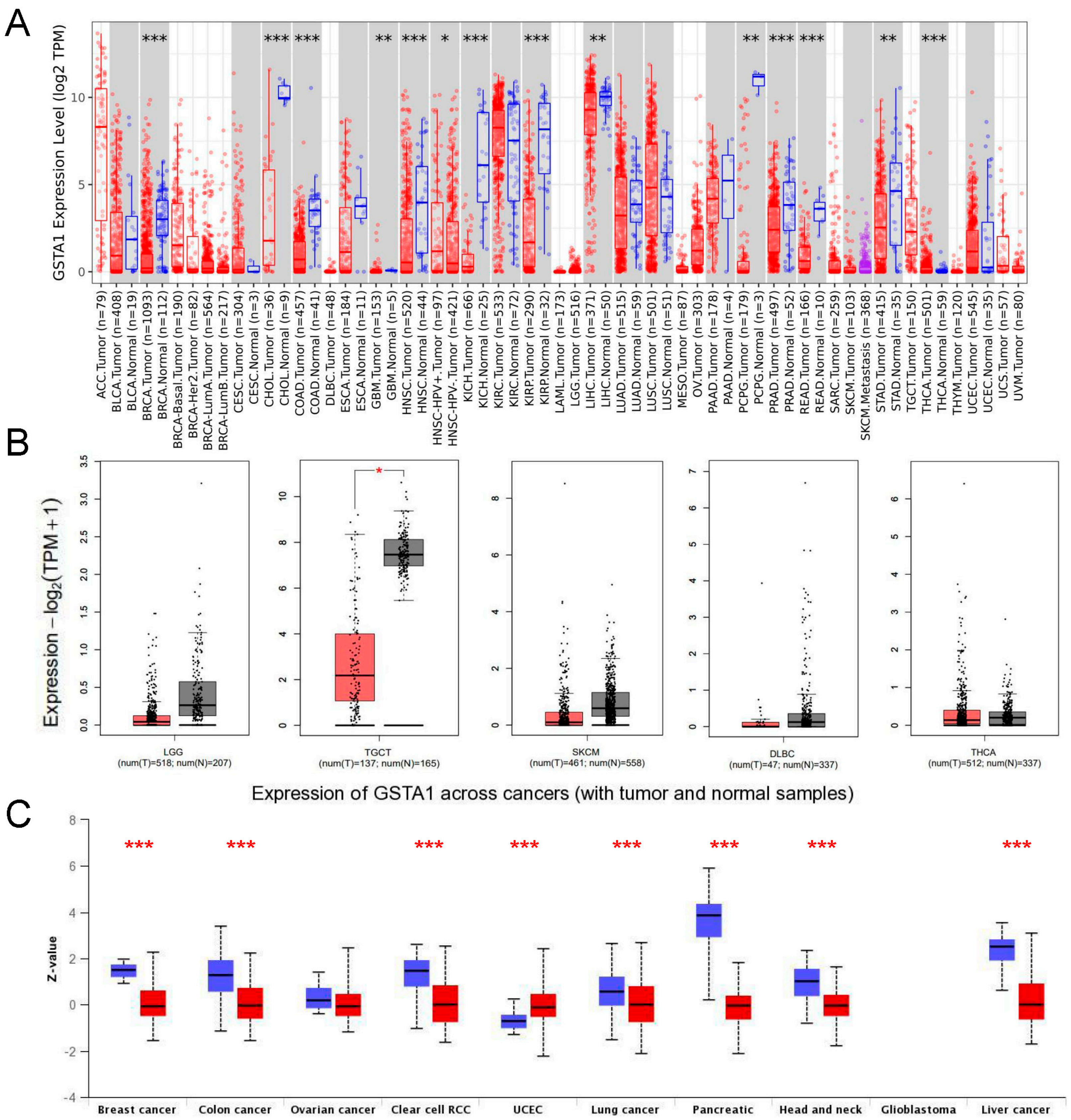


**Figure S4.** The levels of GSTA1 mRNA and protein in different cancers. (**A**,**B**) The mRNA level of GSTA1 in different cancers was analyzed with TIMER2. (**C**) The protein level of GSTA1 in different cancer types was analyzed with UALCAN.

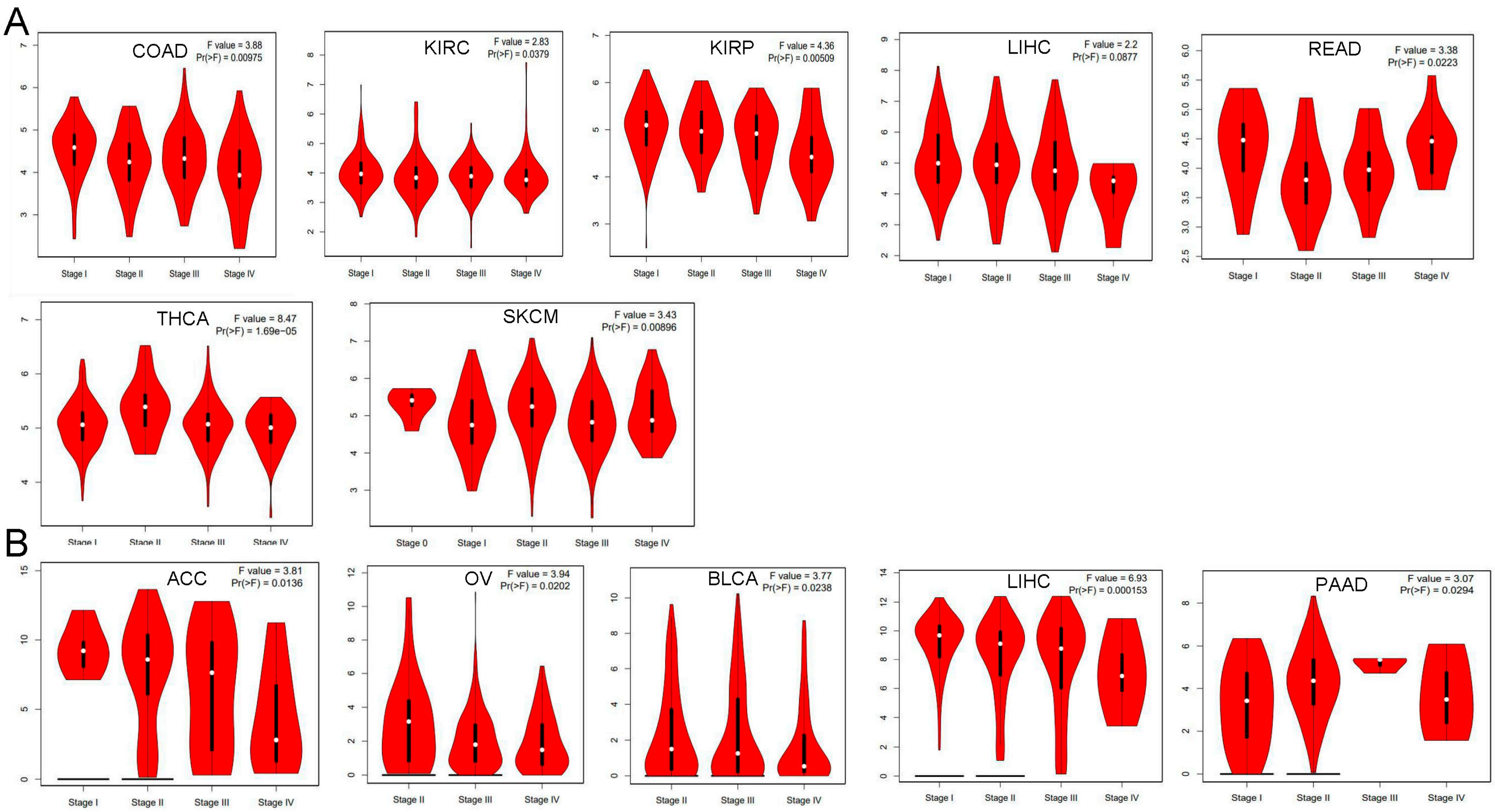


**Figure S5.** Analysis of the TCGA database to investigate correlations between expression of GSTs and cancer prognoses. Based on the TCGA data, the expression levels of the genes encoding GSTZ1 (**A**), and GSTA1 (**B**) were analyzed according to the main pathological stages (stage I, stage II, stage III, and stage IV) of different cancers. The equation $Log_2$(TPM + 1) was applied for the log-scale.

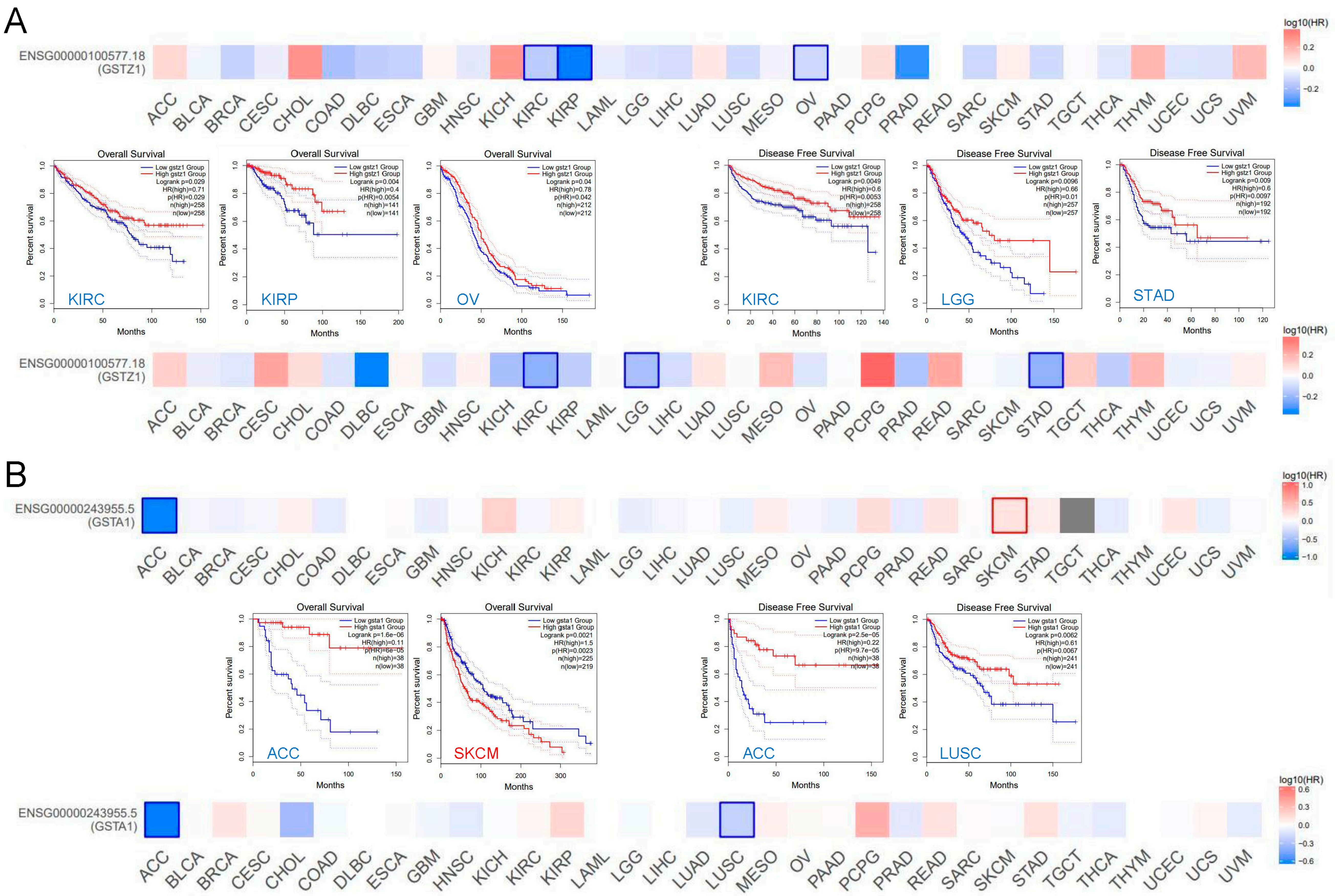


**Figure S6.** Correlation between GSTs expression and cancer prognosis according to the TCGA database. The OS and DFS of different cancers in the TCGA were compared with GSTZ1 expression (**A**) and GSTA1 expression (**B**). The survival map and Kaplan-Meier curves with positive results are given.

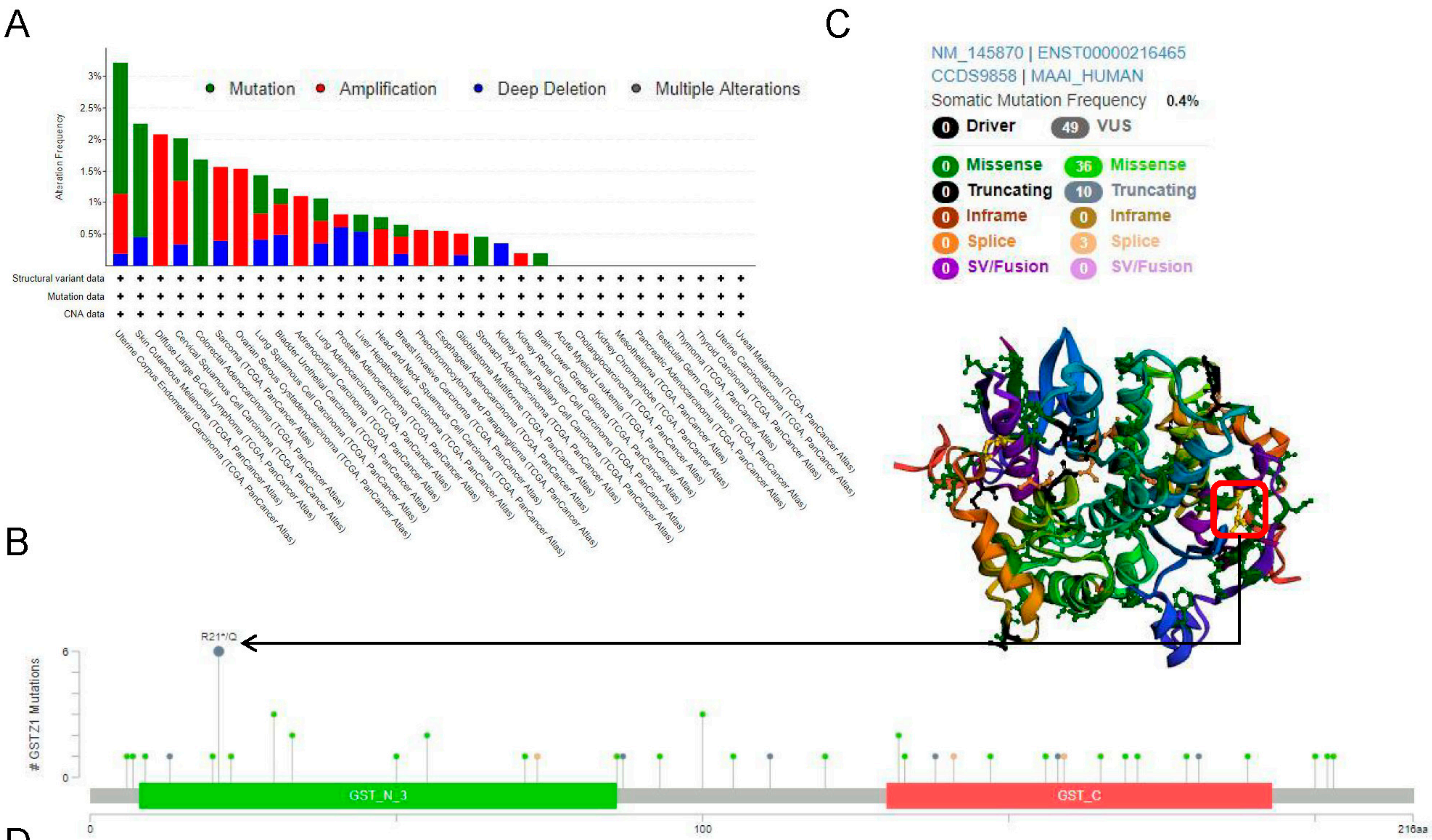


D

| Study of Origin | Sample ID | Cancer Type Detailed | Protein Change | Functional Impact | Mutation Type | Copy # | Start Pos | End Pos | Ref | Var | Allele Freq (T) |
|---|---|---|---|---|---|---|---|---|---|---|---|
| Uterine Corpus Endom... | TCGA-BS-A0UV... | Uterine Endometrioid Carcinoma | R21* | | Nonsense | Diploid | 77791258 | 77791258 | C | T | 0.09 |
| Uterine Corpus Endom... | TCGA-E6-A1LX... | Uterine Endometrioid Carcinoma | R21Q | ● ● | Missense | Diploid | 77791259 | 77791259 | G | A | 0.19 |
| Uterine Corpus Endom... | TCGA-EO-A22... | Uterine Endometrioid Carcinoma | R21Q | ● ● | Missense | Diploid | 77791259 | 77791259 | G | A | 0.29 |
| Colorectal Adenocarcin... | TCGA-F5-6814-01 | Rectal Adenocarcinoma | R21* | | Nonsense | Diploid | 77791258 | 77791258 | C | T | 0.13 |
| Colorectal Adenocarcin... | TCGA-AA-A010... | Colon Adenocarcinoma | R21* | | Nonsense | Diploid | 77791258 | 77791258 | C | T | 0.34 |
| Colorectal Adenocarcin... | TCGA-AZ-4315-... | Colon Adenocarcinoma | R21* | | Nonsense | Diploid | 77791258 | 77791258 | C | T | 0.41 |

**Figure S7.** Mutation of GSTZ1 in different cancers via TCGA analyses. (**A**) The alteration frequency of GSTZ1 with mutation type in various cancers according to the TCGA. (**B**) The alteration frequency of GSTZ1 with mutation site in various cancers according to the TCGA. (**C**,**D**) Information regarding the mutation site with the highest alteration frequency (R21*/Q). This site in the three-dimensional structure of GSTZ1 is shown in yellow.

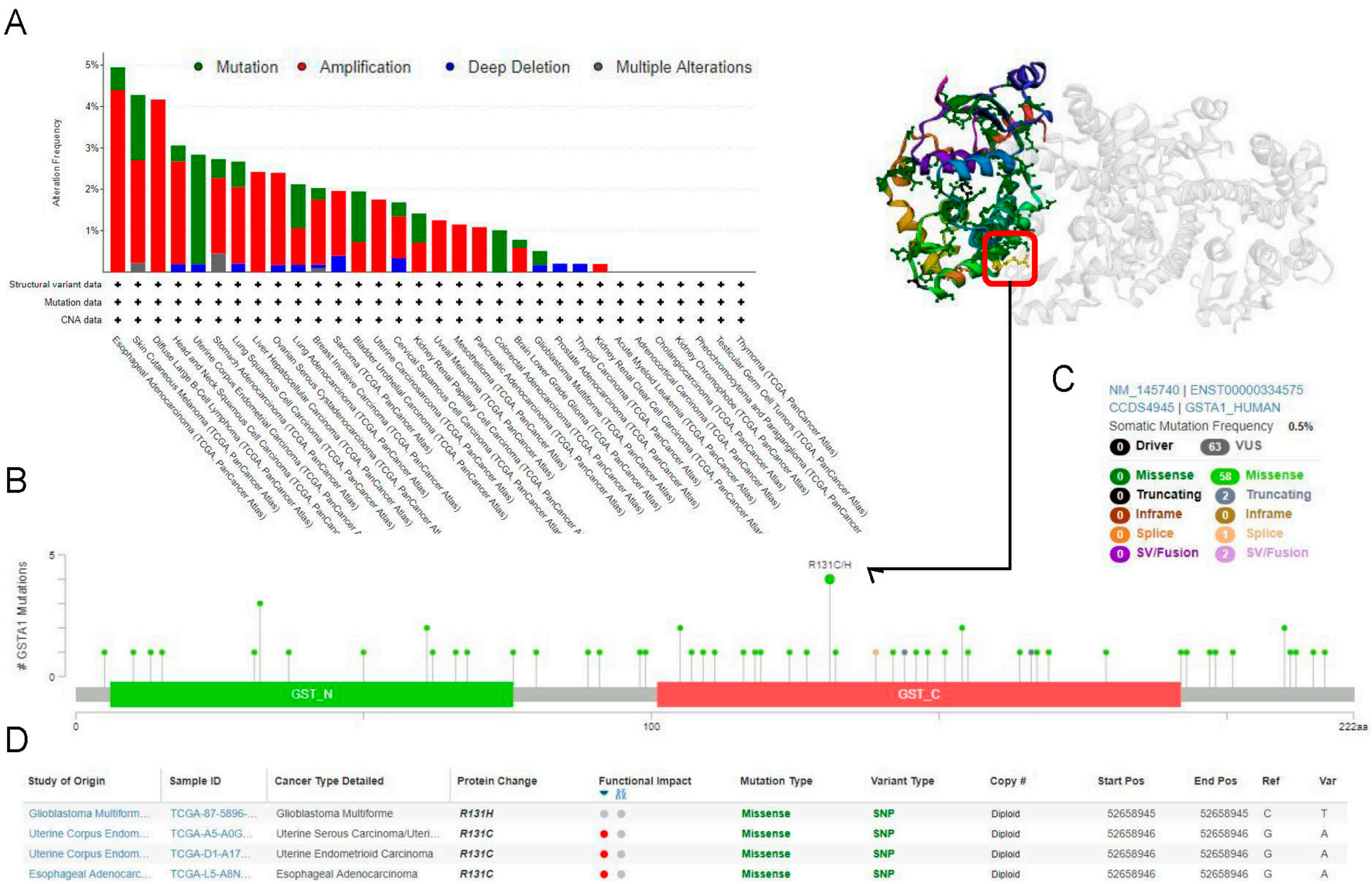


**Figure S8.** Mutation of GSTA1 in different cancers via TCGA analyses. (**A**) The alteration frequency of GSTA1 with mutation type in various cancers according to the TCGA. (**B**) The alteration frequency of GSTA1 with mutation site in various cancers according to the TCGA. (**C**,**D**) Information regarding the mutation site with the highest alteration frequency (R131C/H). This site in the three-dimensional structure of GSTA1 is shown in yellow.

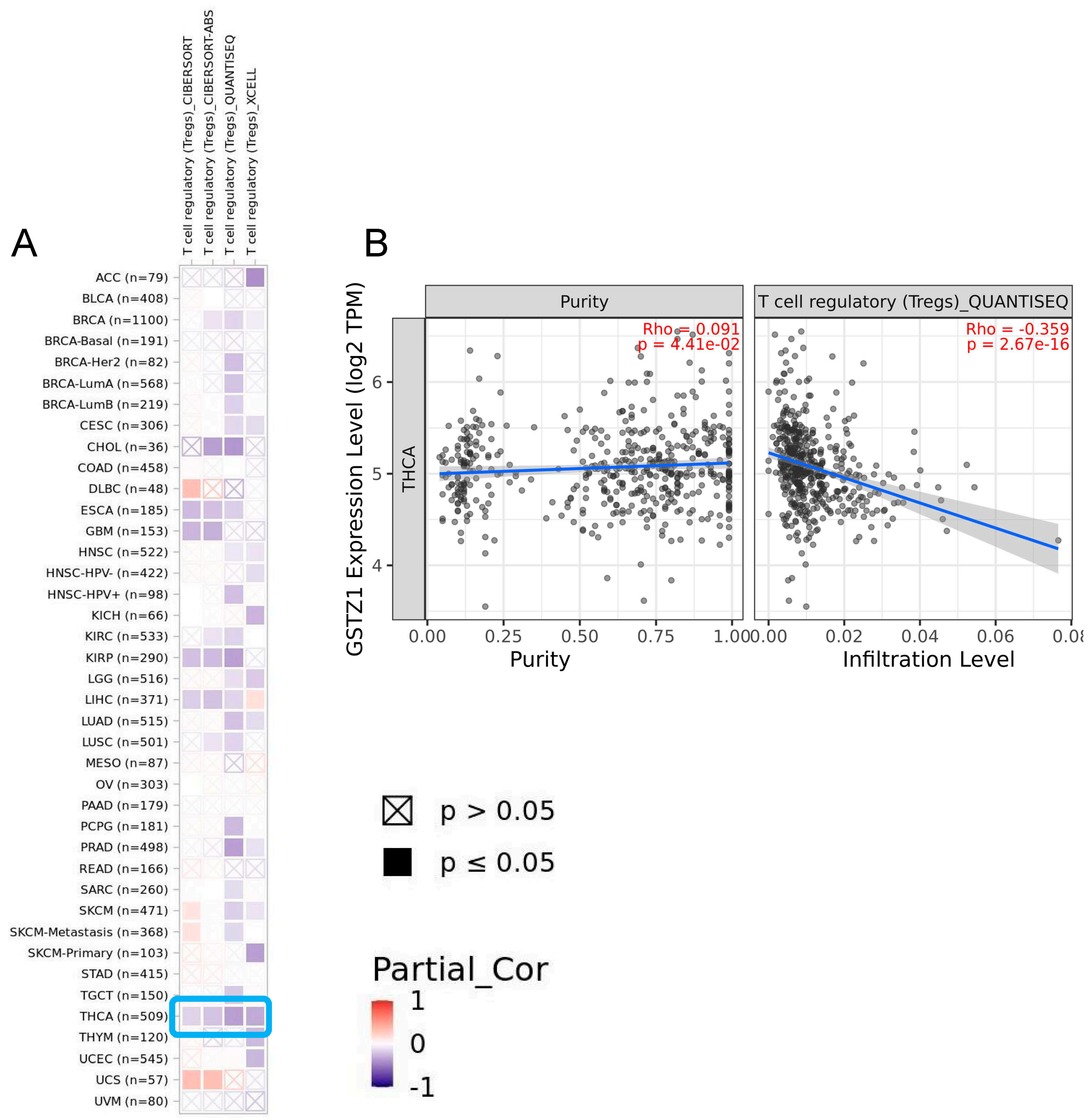


**Figure S9.** Analysis of correlations between GSTZ1 expression and infiltration of cancer-associated immune cells. (**A**) Multiple algorithms were used to explore potential correlations between the expression of GSTZ1 and the infiltration of cancer-associated Tregs across all types of cancer in TCGA. (**B**) The results with the highest correlation scores, which were verified by multiple algorithms, are shown.

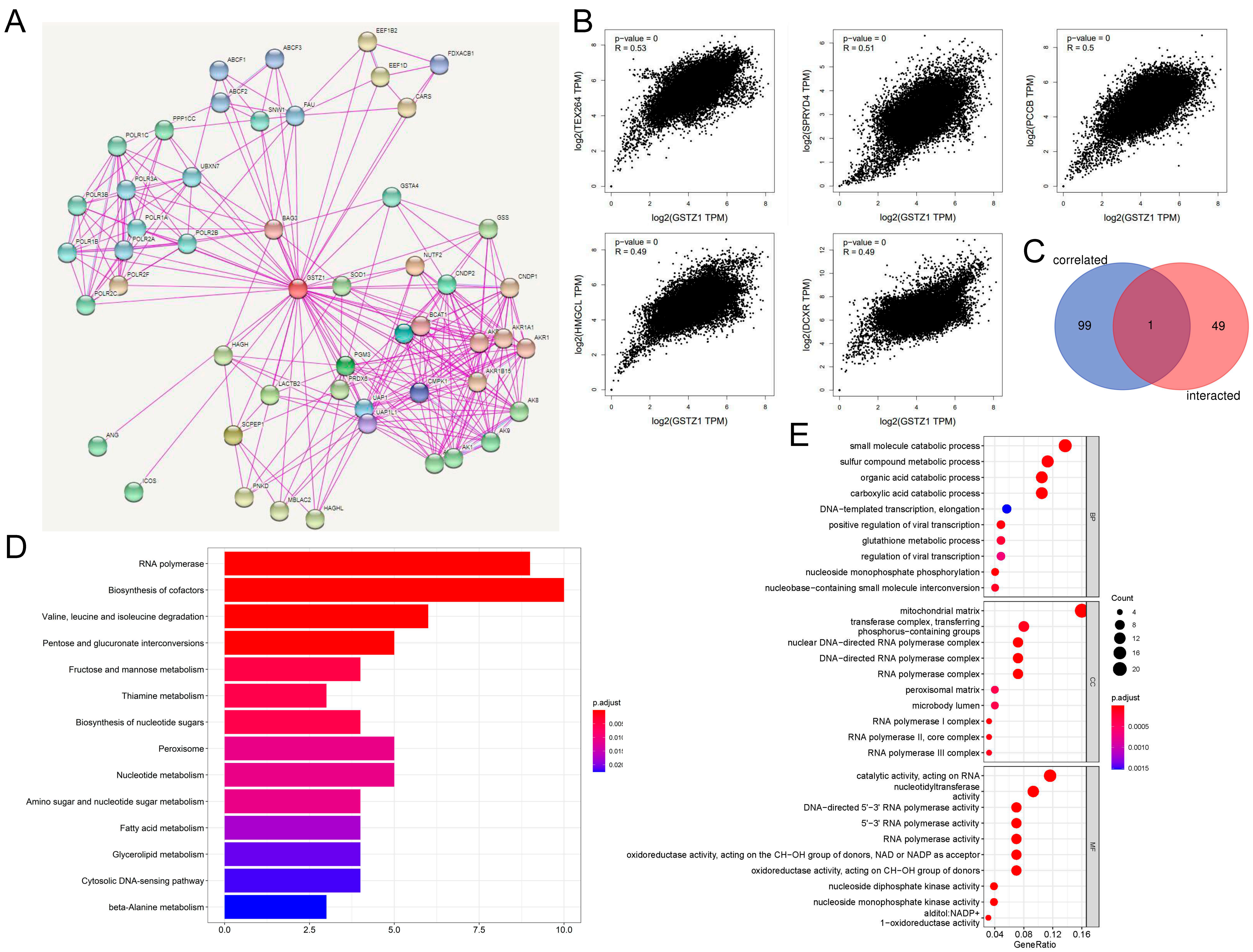

**Figure S10.** GSTZ1-related gene enrichment analysis. (**A**) Experimentally determined GSTZ1-binding proteins were identified using STRING. (**B**) The correlations of the expression of GSTZ1 and the five most related genes in cancers are shown. (**C**) An analysis of intersections of genes encoding GSTZ1-binding proteins and expression-correlated genes was conducted. (**D**) KEGG pathway analyses were performed on the genes encoding GSTZ1-binding proteins and expression-correlated genes. (**E**) GO analyses were also performed.

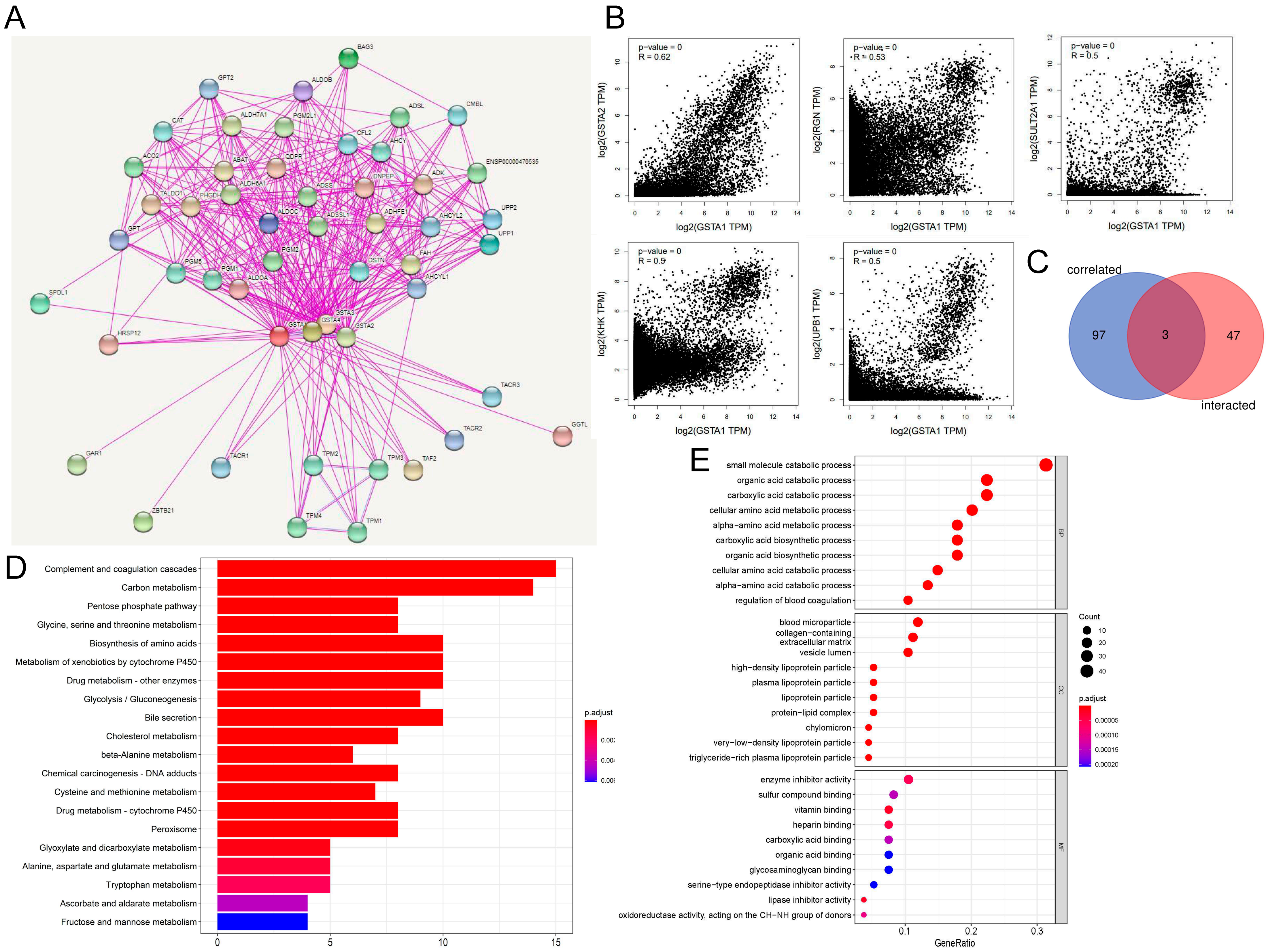

**Figure S11.** GSTA1-related gene enrichment analysis. (**A**) Experimentally determined GSTA1-binding proteins were identified using STRING. (**B**) The correlations of the expression of GSTA1 and the five most related genes in cancers are shown. (**C**) An analysis of intersections of genes encoding GSTA1-binding proteins and expression-correlated genes was conducted. (**D**) KEGG pathway analyses were performed on the genes encoding GSTA1-binding proteins and expression-correlated genes. (**E**) GO analyses were also performed.

A

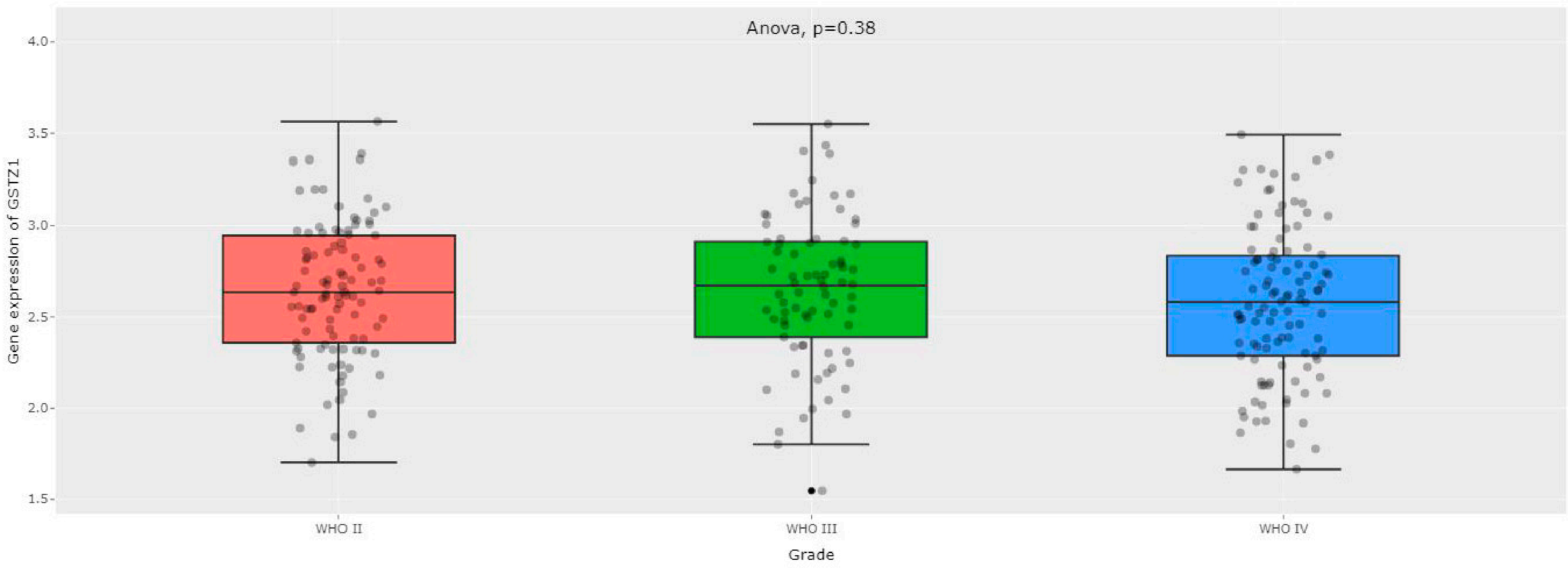


B

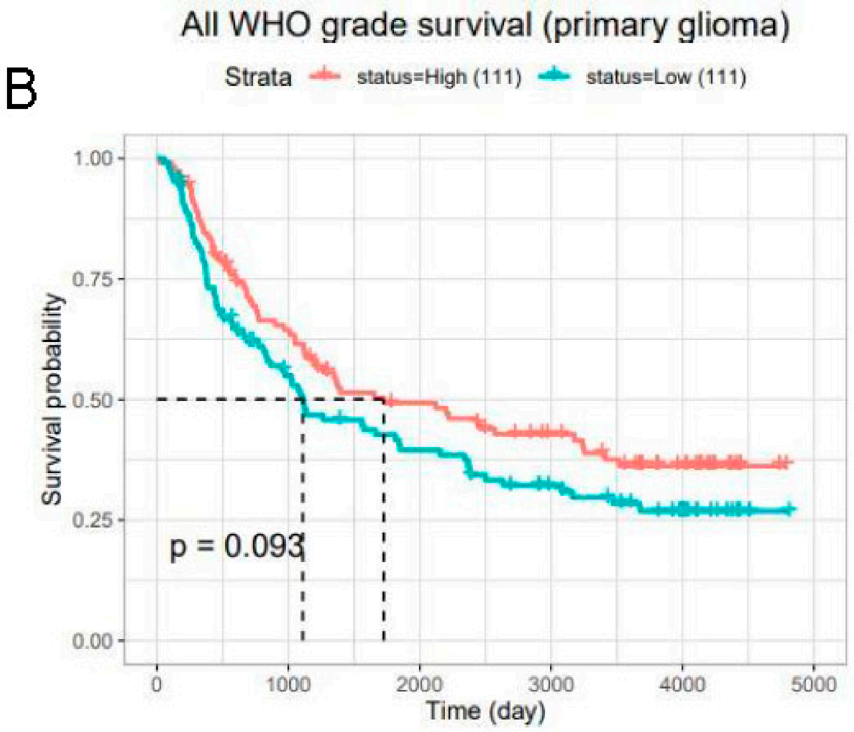


**Figure S12.** Correlation analysis of GSTZ1 expression and patient prognosis based on the CGGA database. (**A**) The results of CGGA database analyses showed the level of GSTZ1 expression in glioma. (**B**) Differences in survival rates of patients according to GSTZ1 expression levels did not reach statistical significance in all glioma patients.

| Abbreviation | Full name |
| --- | --- |
| ACC | Adrenocortical carcinoma |
| BLCA | Bladder Urothelial Carcinoma |
| BRCA | Breast invasive carcinoma |
| CESC | Cervical squamous cell carcinoma and endocervical adenocarcinoma |
| CHOL | Cholangio carcinoma |
| COAD | Colon adenocarcinoma |
| DLBC | Lymphoid Neoplasm Diffuse Large B-cell Lymphoma |
| ESCA | Esophageal carcinoma |
| GBM | Glioblastoma multiforme |
| HNSC | Head and Neck squamous cell carcinoma |
| KICH | Kidney Chromophobe |
| KIRC | Kidney renal clear cell carcinoma |
| KIRP | Kidney renal papillary cell carcinoma |
| LAML | Acute Myeloid Leukemia |
| LGG | Brain Lower Grade Glioma |
| LIHC | Liver hepatocellular carcinoma |
| LUAD | Lung adenocarcinoma |
| LUSC | Lung squamous cell carcinoma |
| MESO | Mesothelioma |
| OV | Ovarian serous cystadenocarcinoma |
| PAAD | Pancreatic adenocarcinoma |
| PCPG | Pheochromocytoma and Paraganglioma |
| PRAD | Prostate adenocarcinoma |
| READ | Rectum adenocarcinoma |
| SARC | Sarcoma |
| SKCM | Skin Cutaneous Melanoma |
| STAD | Stomach adenocarcinoma |
| TGCT | Testicular Germ Cell Tumors |
| THCA | Thyroid carcinoma |
| THYM | Thymoma |
| UCEC | Uterine Corpus Endometrial Carcinoma |
| UCS | Uterine Carcinosarcoma |
| UVM | Uveal Melanoma |

**Figure S13.** Abbreviations and full names of each cancer mentioned in this manuscript.
